# Multiple serum anti-glutamate receptor antibody levels in clozapine-treated/naïve patients with treatment-resistant schizophrenia

**DOI:** 10.1186/s12888-024-05689-0

**Published:** 2024-04-02

**Authors:** Jingqi He, Jinguang Li, Yisen Wei, Zhangyin He, Junyu Liu, Ning Yuan, Risheng Zhou, Xingtao He, Honghong Ren, Lin Gu, Yanhui Liao, Xiaogang Chen, Jinsong Tang

**Affiliations:** 1 Department of Psychiatry, Sir Run-Run Shaw Hospital, School of Medicine, Zhejiang University, Hangzhou, China; 2 Department of Psychiatry, National Clinical Research Center for Mental Disorders, and National Center for Mental Disorders, The Second Xiangya Hospital of Central South University, Changsha, China; 3 Affiliated Wuhan Mental Health Center, Tongji Medical College, Huazhong University of Science and Technology, Wuhan, China; 4 Department of Psychiatry, Jiangxi Provincial People’s Hospital, The First Affiliated Hospital of Nanchang Medical College, Nanchang, China; 5 School of Mental Health, Wenzhou Medical University, Wenzhou, China; 6 Xiangya Nursing School of Central South University, Changsha, China; 7 Hunan Provincial Brain Hospital (The second people’s Hospital of Hunan Province), Changsha, China; 8 The Ninth Hospital of Changsha, Changsha, China; 9 Department of Psychiatry, Shandong Provincial Hospital, Shandong University, Jinan, China; 10 RIKEN Center for Advanced Intelligence Project, Tokyo, Japan; 11 Research Center for Advanced Science and Technology (RCAST), University of Tokyo, Tokyo, Japan; 12 Zigong Mental Health Center, Zigong, China

**Keywords:** Treatment-resistant schizophrenia, Clozapine, Anti-glutamate receptor antibody, NMDA, AMPA, mGluR

## Abstract

**Background:**

Glutamatergic function abnormalities have been implicated in the etiology of treatment-resistant schizophrenia (TRS), and the efficacy of clozapine may be attributed to its impact on the glutamate system. Recently, evidence has emerged suggesting the involvement of immune processes and increased prevalence of antineuronal antibodies in TRS. This current study aimed to investigate the levels of multiple anti-glutamate receptor antibodies in TRS and explore the effects of clozapine on these antibody levels.

**Methods:**

Enzyme linked immunosorbent assay (ELISA) was used to measure and compare the levels of anti-glutamate receptor antibodies (NMDAR, AMPAR, mGlur3, mGluR5) in clozapine-treated TRS patients (TRS-C, *n* = 37), clozapine-naïve TRS patients (TRS-NC, *n* = 39), and non-TRS patients (nTRS, *n* = 35). Clinical symptom severity was assessed using the Positive and Negative Symptom Scale (PANSS), while cognitive function was evaluated using the MATRICS Consensus Cognitive Battery (MCCB).

**Result:**

The levels of all four glutamate receptor antibodies in TRS-NC were significantly higher than those in nTRS (*p* < 0.001) and in TRS-C (*p* < 0.001), and the antibody levels in TRS-C were comparable to those in nTRS. However, no significant associations were observed between antibody levels and symptom severity or cognitive function across all three groups after FDR correction.

**Conclusion:**

Our findings suggest that TRS may related to increased anti-glutamate receptor antibody levels and provide further evidence that glutamatergic dysfunction and immune processes may contribute to the pathogenesis of TRS. The impact of clozapine on anti-glutamate receptor antibody levels may be a pharmacological mechanism underlying its therapeutic effects.

## Background

Approximately 30% of individuals with schizophrenia do not respond to antipsychotic medications, which is known as treatment-resistant schizophrenia (TRS) [1]. TRS is typically defined as schizophrenia that shows inadequate response to at least two different antipsychotics despite adequate treatment according to various guidelines and clinical trials [2, 3]. The underlying pathophysiological mechanisms of TRS remain elusive. The categorical hypothesis suggests that the pathophysiology of TRS may differ significantly from non-treatment-resistant schizophrenia (nTRS), making conventional antipsychotics ineffective [4]. Previous studies have demonstrated that TRS is associated with deficits in glutamatergic function [5, 6]. Among available treatments, clozapine is the only effective option for TRS. While the exact mechanisms of its therapeutic effects are still not fully understood, emerging evidence suggests that clozapine may exert its efficacy through modulation of the glutamatergic system, in contrast to most other antipsychotics that primarily act through dopamine-2 receptor blockade [7, 8]. Positron emission tomography (PET) studies have shown that there are some patients who have limited response to high levels of dopamine receptor blockade, and reduced dopamine levels were not effective for TRS [9, 10]. Furthermore, proton magnetic resonance spectroscopy (1H-MRS) contributes to better observation of glutamatergic systems dysfunction of TRS. Previous studies have shown that glutamate levels in the anterior cingulate cortex (ACC) in TRS are higher than in nTRS, while no group differences were found in striatal dopamine function [11]. Hence, according to the categorical hypothesis, TRS may be a specific subtype of schizophrenia, which is more related to the dysfunction of the glutamatergic system rather than the dopaminergic system.

Additionally, complex interactions between the immune system and the brain have been implicated in the pathophysiology of neuropsychiatric disorders [12]. In addition to neurotransmitter-based hypotheses such as the glutamatergic hypothesis, there is evidence suggesting immune dysregulation in schizophrenia [7]. Lin’s study in 1998 first linked TRS to activation of the inflammatory response system, showing significantly elevated serum IL-6 levels in TRS compared to controls and nTRS [13]. Subsequent studies have also reported increased levels of SIL-1RA, IL-2, IL-10, STNF-R1, STNF-R2, IL-8, chemokine ligand (CCL)-3, and CCL-2 in TRS [14–18].

Neurotransmitters and the immune system are not isolated and independent, but they interact with each other, and antineuronal antibodies can serve as a bridge connecting these two systems [19]. On one hand, autoimmune diseases have been identified as risk factors for schizophrenia [20]. On one hand, autoimmune diseases have been identified as risk factors for schizophrenia [21]. Bechter proposed the “mild encephalitis hypothesis,” suggesting that certain severe mental disorders, such as schizophrenia, may be attributed to chronic low-level neuroinflammation [22]. In 2007, Dalmau et al. first described autoimmune encephalitis associated with antibodies against NMDA-type glutamate receptors [23, 24]. Aside from neurological symptoms, psychiatric symptoms, and cognitive impairment are also common features of autoimmune encephalitides [25]. In some reported cases, patients may initially present with isolated psychiatric episodes [26, 27]. These findings suggest the potential involvement of glutamate receptor antibodies in the pathophysiology of schizophrenia. Previous studies have demonstrated that blood-brain barrier (BBB) integrity is compromised in individuals with schizophrenia, leading to increased permeability [28, 29]. As a result, circulating glutamate receptor antibodies may penetrate the damaged BBB, bind to their corresponding receptors in the central nervous system, disrupt glutamate receptor function, and ultimately contribute to the manifestation of psychiatric symptoms and cognitive impairment.

Several studies have investigated the prevalence and serum levels of glutamate receptor antibodies in individuals with schizophrenia. Previous studies primarily focused on NMDA receptor antibodies and utilized immunofluorescence as the detection method [25, 30, 31]. However, the results from these studies are qualitative and have yielded mixed findings. In 2019, Tong et al. conducted the first study to employ enzyme-linked immunosorbent assay (ELISA) for detecting NMDAR antibody levels in patients with schizophrenia [32]. They observed elevated serum NMDA receptor antibody levels in first-episode schizophrenia patients compared to healthy controls, which were also correlated with symptom severity. Subsequently, ELISA was further utilized in two additional studies by the same group. They revealed significantly higher serum NMDA receptor antibody levels in patients with treatment-resistant schizophrenia compared to those with non-treatment-resistant schizophrenia, which were also associated with white matter deficits [33]. Another study demonstrated elevated antibody levels in schizophrenic patients with tardive dyskinesia [34].

Autoantibodies targeting glutamate receptors offer a valuable means to connect the two fundamental hypotheses regarding the pathogenesis of schizophrenia, namely the glutamatergic dysfunction hypothesis and the neuroimmunological hypothesis [19]. Moreover, most current studies investigating the association between glutamate receptors and schizophrenia have primarily focused on the NMDA-type receptor, while the involvement of other types of glutamate receptors in the pathophysiology of schizophrenia has gained increasing attention in recent years. Additionally, a speculation has emerged suggesting that TRS may represent a population enriched for the presence of anti-neuronal antibodies [35]. Furthermore, it has been discovered that clozapine can induce secondary antibody deficiency (SAD) [36, 37]. Consequently, researchers have proposed the possibility that clozapine exerts its therapeutic effects in TRS by reducing anti-glutamate receptor antibody levels [37]. Given the limited number of studies exploring the relationship between TRS, glutamate receptor antibodies, and clozapine, this study aimed to investigate multiple glutamate receptor antibody levels in TRS patients with and without clozapine treatment. We hypothesized that patients with TRS may exhibit elevated antibody levels and that clozapine can ameliorate clinical symptoms and cognitive impairment by reducing these levels and upregulating glutamate receptor function.

## Materials and methods

### Participants

Participants were recruited from the Second Xiangya Hospital of Central South University from 2020 to 2022. The inclusion criteria required that participants were right-handed, aged between 18 and 50, and met the Diagnostic and Statistical Manual of Mental Disorders, Fifth Edition, criteria for schizophrenia. The exclusion criteria for all participants were (1) substance abuse, (2) a history of head trauma resulting in loss of consciousness, or (3) major medical or neurological illness.

We reviewed the medical records of all patients and classified TRS patients versus nTRS patients based on their previous responses to antipsychotics. According to the consensus guidelines published by the Treatment Response and Resistance to Psychosis (TRRIP) Working Group in 2017, TRS was defined as schizophrenia that had poor response (Positive and Negative Syndrome Scale>60, Global Assessment of Functioning ≤60) to two different adequate doses (dosage equivalence of chlorpromazine ≥600 mg/day) and courses (≥6 weeks) of non-clozapine antipsychotics [3]. To make sure that after adequate treatment patients still had disabling symptoms due to resistance to antipsychotics, we also excluded patients due to intolerance to antipsychotics. Although clozapine is recommended as the gold standard treatment for TRS in all clinical guidelines, its use is frequently declined by patients and their guardians in clinical practice due to its potential side effects. As a result, these patients often continue maintenance treatment with other atypical antipsychotic medications instead of using clozapine as monotherapy. Therefore, we further divided TRS patients into two groups according to whether clozapine was applied (≥ 200 mg/day， ≥ 12-week duration) or not. Ultimately, a total of 111 patients were included and divided into three groups: clozapine-treated patients with TRS (TRS-C), clozapine-naïve patients with TRS (TRS-NC) and clozapine-naïve patients with nTRS (nTRS). This study was approved by the Second Xiangya Hospital Ethics Committee (No. S006, 2018), and all participants fully understood the research procedures and provided written informed consent.

### Clinical evaluation

Clinical evaluation was performed independently by two experienced senior psychiatrists. The severity of illness was assessed by the Positive and Negative Syndrome Scale (PANSS) and Global Assessment of Functioning scale (GAF). Neurocognition assessment instrument was the Chinese version of the MATRICS Consensus Cognitive Battery (MCCB), which contains a total of seven domains: (1) Speed of processing; (2) Attention/vigilance; (3) Working memory; (4) Verbal learning; (5) Visual learning; (6) Reasoning and problem solving and; (7) Social cognition.

### Measurement of anti-glutamate receptor antibodies

On the day of enrollment or the next morning, fasting venous blood was collected and placed in a non-anticoagulant vacuum tube. The blood samples were rested and centrifuged (3000 rpm for 10 minutes), and the serum was extracted and stored in an ultra-low temperature refrigerator (− 80 °C). Serum anti-NMDAR antibody, anti-AMPAR antibody, anti-mGluR3 antibody, and anti-mGluR5 antibody levels were measured by double antigen sandwich ELISA method using commercially available kits (FANKEW, Shanghai, China). Each sample was tested twice, and the final results were averaged from the two assay values. Intra-and inter-assay variation coefficients of anti-NMDAR antibody were < 4.5 and < 6.7%; intra-and inter-assay variation coefficients of anti-AMPAR antibody were < 5.0 and < 7.5%; intra-and inter-assay variation coefficients of anti-mGluR3 antibody were < 4.2 and < 6.4%; intra-and inter-assay variation coefficients of anti-mGluR5 antibody were < 4.4 and < 6.6%, respectively.

### Statistical analysis

All statistical analyses were performed using SPSS (version 25 IBM Inc. New York, USA). Means ± standard deviations (X ± S) were used for measurement data. A two-tailed *p* value < 0.05 indicated statistical significance. After obtaining MCCB row scores, standard score-T score conversion was performed. One-way ANOVA or chi-square test was used to statistically analyze the demographic data such as gender, age, PANSS score, duration of disease, medication dose, and MCCB score among the three groups. The Shapiro-Wilk test showed that the levels of various anti-glutamate receptor antibodies in the three groups did not fall into a normal distribution; therefore, Spearman correlations were used to analyze the antibody levels with PANSS scores, MCCB scores, and clozapine dose. Due to the differences in demographic and clinical characteristics and their potential associations with antibody levels, we included years of education, duration of illness, medication, PANSS total scores, MCCB scores as covariates, and the generalized linear model (GLM) was used to compare the differences in antibody levels among the groups. FDR correction was performed for multiple comparisons.

## Results

### Demographic and clinical characteristics

Thirty-seven patients with TRS-C, 39 patients with TRS-NC and 35 patients with nTRS were included in this study. The demographic and clinical characteristics of the participants are summarized in Table 1. There were no significant differences in gender composition and age among the three groups. After post hoc comparison, it was found that the years of education in the nTRS group were higher than those in the TRS-NC (*p* = 0.014) and TRS-C groups (*p* = 0.002); the duration of disease in the TRS-C group was longer than that in the nTRS group (*p* = 0.005); the dose of medication in the TRS-NC group was greater than that in the nTRS group (*p* = 0.007). Due to the differences in years of education, course of disease, and dosage of medication among the three groups, we further conducted correlation analyses between these indicators and serum glutamate receptor antibody levels in the following.

**Table 1:** Demographic and clinical characteristics and antibody levels of clozapine-treated patients with TRS, clozapine-naïve patients with TRS and clozapine-naïve patients with nTRS

	nTRS (*n* = 35)	TRS-NC (*n* = 39)	TRS-C (*n* = 37)	Statistic F/χ 2/ Wald χ^2^ (*p*-value)	*p-value* nTRS vs TRS-NC	*p-value* nTRS vs TRS-C	*p-value* TRS-NC vs TRS-C
**Demographic**							
Gender(M/F)	16/19	18/21	19/18	0.29(0.865)	0.970	0.632	0.650
Age (years)	30.37 ± 9.08	30.54 ± 8.59	32.65 ± 10.45	0.513(0.513)	1.000	0.919	0.990
Education (years)	12.73 ± 2.91	10.79 ± 2.97	10.30 ± 2.77	7.10(0.001**)	0.014*	0.002**	1.000
**Clinical**							
Duration of illness (years)	7.54 ± 6.67	10.13 ± 6.96	13.51 ± 9.55	5.26(0.007**)	0.479	0.005**	0.188
Medication (CPZE mg/day)	447.00 ± 223.51	620.96 ± 232.79	534.46 ± 242.52	5.00(0.008**)	0.007**	0.344	0.377
Clozapine dose (mg/day)	–	–	270.27 ± 82.04	–	–	–	–
PANSS Positive	13.23 ± 5.17	22.95 ± 6.64	18.57 ± 7.44	20.58(< 0.001***)	< 0.001***	0.002**	0.012*
PANSS Negative	11.29 ± 4.39	20.74 ± 8.85	20.30 ± 8.04	F = 18.59(< 0.001***)	< 0.001***	< 0.001***	1.000
PANSS General	22.60 ± 4.60	38.05 ± 7.66	35.32 ± 8.13	49.90(< 0.001***)	< 0.001***	< 0.001***	0.281
PANSS Total	46.97 ± 9.12	82.79 ± 13.57	73.95 ± 17.31	66.26(< 0.001***)	< 0.001***	< 0.001***	0.019*
GAF	67.51 ± 7.14	44.03 ± 10.95	52.65 ± 16.91	33.63(< 0.001***)	< 0.001***	< 0.001***	0.009**
Speed of processing	35.86 ± 8.00	24.97 ± 11.46	31.97 ± 9.93	11.44(< 0.001***)	< 0.001***	0.303	0.008**
Attention/vigilance	38.23 ± 11.12	27.49 ± 11.04	28.92 ± 12.82	8.99(< 0.001***)	< 0.001***	0.003**	1.000
Working memory	37.83 ± 10.45	27.03 ± 10.77	34.81 ± 12.64	9.07(< 0.001***)	< 0.001***	0.784	0.010*
Verbal learning	32.63 ± 10.45	26.85 ± 9.34	27.76 ± 9.30	3.72(0.027*)	0.035*	0.106	1.000
Visual learning	41.34 ± 13.75	23.77 ± 11.64	26.22 ± 13.69	19.29(< 0.001***)	< 0.001***	< 0.001***	1.000
Reason and problem solving	37.37 ± 11.20	32.77 ± 12.13	34.22 ± 12.42	1.42(0.247)	0.303	0.795	1.000
Social cognition	41.89 ± 15.00	31.08 ± 10.22	38.59 ± 10.19	8.08(0.001**)	0.001**	0.733	0.021*
MCCB total score	37.43 ± 6.80	26.31 ± 8.70	31.97 ± 7.91	18.42(< 0.001***)	< 0.001***	0.012*	0.007**
**Antibody levels**							
NMDAR Ab (pg/ml)	188.79 ± 4.05	269.37 ± 3.83	203.27 ± 3.84	137.48(< 0.001***)	< 0.001***	0.638	< 0.001***
AMPAR Ab (pg/ml)	1630.76 ± 30.43	2098.86 ± 28.80	1803.85 ± 28.92	56.14(< 0.001***)	< 0.001***	0.032*	< 0.001***
mGluR3 Ab (pg/ml)	87.03 ± 2.77	114.26 ± 2.62	82.47 ± 2.63	67.77(< 0.001***)	< 0.001***	0.105	< 0.001***
mGluR5 Ab (pg/ml)	56.71 ± 1.41	81.91 ± 1.08	61.48 ± 1.08	174.45(< 0.001***)	< 0.001***	0.502	< 0.001***

Values are provided as mean ± SD unless otherwise stated. *PANSS* Positive and Negative Syndrome Scale, *GAF* Global Assessment of Functioning scale, *CPZE* chlorpromazine equivalent dose, *MCCB* MATRICS Consensus Cognitive Battery, *Ab* antibody

*p < .05, **p < .01, ***p < .001

The two TRS groups had higher illness severity (i.e. PANSS total and subscale scores, as well as GAF scores) compared to the NTRS group. Compared TRS-NC patients, TRS-C patients had lower total PANSS (*p* = 0.019) and PANSS positive sub-scores (*p* = 0.012), and higher GAF scores (*p* = 0.009). There were no significant differences in PANSS-negative sub-scores and general sub-scores between the two TRS groups.

Except for the reasoning and problem-solving domain, significant differences were observed among the three groups in the six sub-items and the total scores of MCCB. Post hoc comparisons revealed that the nTRS group had higher scores than the TRS-NC group in the other six sub-items (all ps < 0.05), as well as in attention/vigilance (*p* = 0.003) and visual learning (*p* < 0.001) compared to the TRS-C group. The TRS-C group showed significantly higher scores than the TRS-NC group in speed of processing (*p* = 0.008), working memory (*p* = 0.010), and social cognition (*p* = 0.021). Furthermore, the nTRS group obtained the highest total score, followed by the two TRS groups. The nTRS group had a significantly higher total score than the TRS-C group (*p* = 0.012), and the TRS-C group had a significantly higher total score than the TRS-NC group (*p* = 0.007) (Table 1).

### Group comparisons of anti-glutamate receptor antibody levels

The levels of the four anti-glutamate receptor antibodies were compared between the three groups using a Generalized Linear Model (GLM) with years of education, duration of illness, medication, PANSS total scores, MCCB scores included as covariates. All the above comparisons were adjusted using FDR correction. There were no main effects of the covariates was reported.

**Fig. 1:**
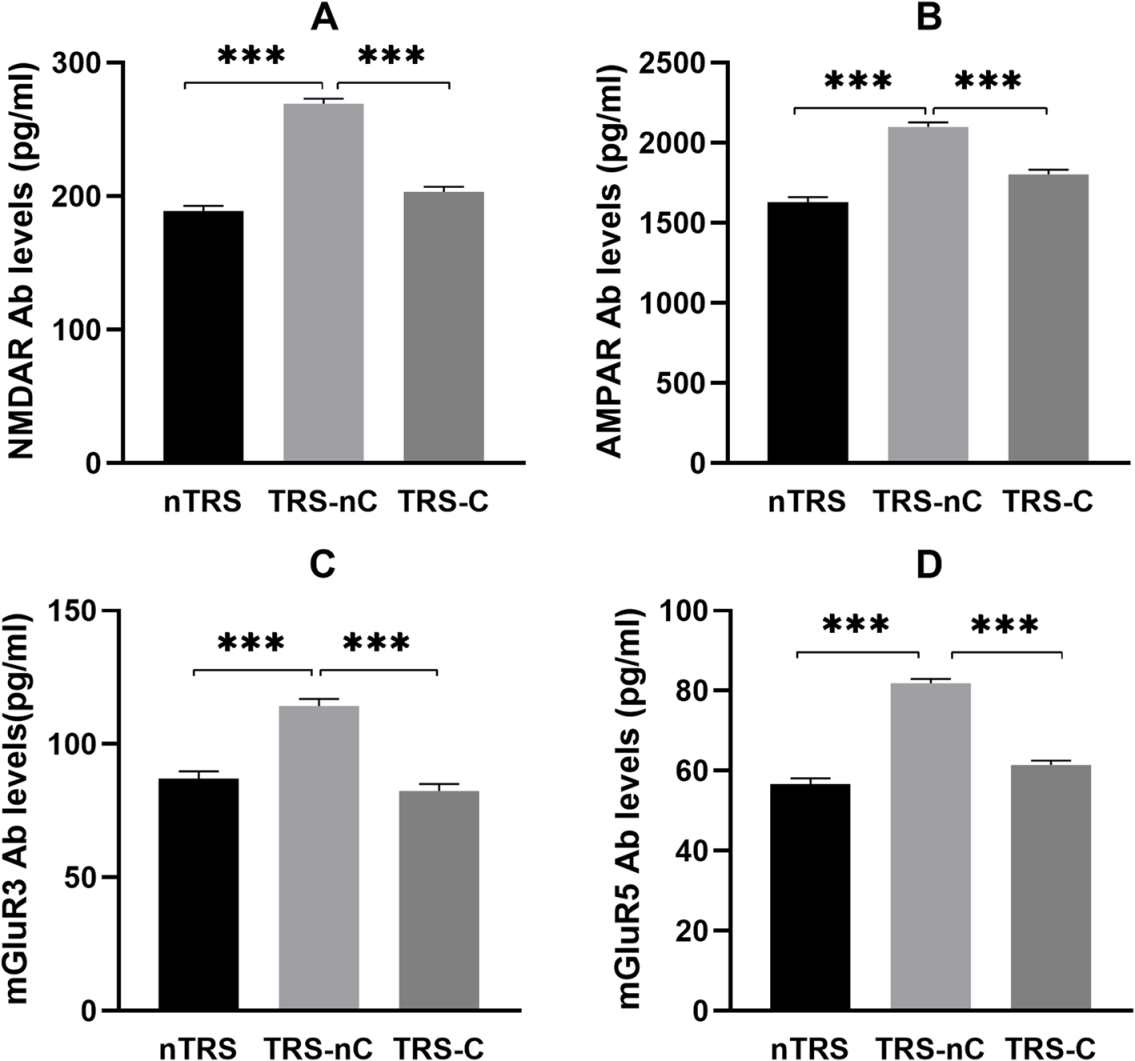
Serum anti-glutamate receptor antibody levels (mean and SD) of TRS-C, TRS-nC and nTRS. Abbreviations: TRS-C, clozapine-naïve patients with treatment resistant schizophrenia; TRS-nC, clozapine-naïve patients with non-treatment resistant schizophrenia; nTRS, clozapine-naïve patients with non-treatment resistant schizophrenia; NMDAR, N-methyl-D-aspartate Receptor; AMPAR, Alpha-amino-3-hydroxy-5-methylisoxazole-4-propionic acid receptor; mGluR, Metabotropic glutamate receptor; Ab, antibody. ***p < .001

The anti-NMDAR antibody levels were significantly higher in the TRS-NC group than in the TRS-C group (*p* < 0.001) and the nTRS group (*p* < 0.001), and there was no significant difference between the TRS-C and nTRS groups (*p* = 0.638) (Table 1, Fig. 1A).

The anti-AMPAR antibody levels were significantly higher in the TRS-NC group than in the other two groups (ps < 0.001). The levels of the TRS-C group were higher than of the nTRS group before the correction (*p* < 0.032, FDR-corrected *q* = 0.128 > 0.05) (Table 1, Fig. 1B).

The anti-mGluR3 antibody levels in the TRS-NC group were significantly higher than that in the other two groups (ps < 0.001), and there was no significant difference between the TRS-C and nTRS groups (*p* = 0.105) (Table 1, Fig. 1C).

Similar to the other antibodies, the TRS-NC group had the highest anti-mGluR5 antibody levels compared with the other two groups (p’s < 0.001), while there was no significant difference between the TRS-C and nTRS groups (*p* = 0.502) (Table 1, Fig. 1D).

### Relationship between anti-glutamate receptor antibody levels and symptom severity

**Fig. 2:**
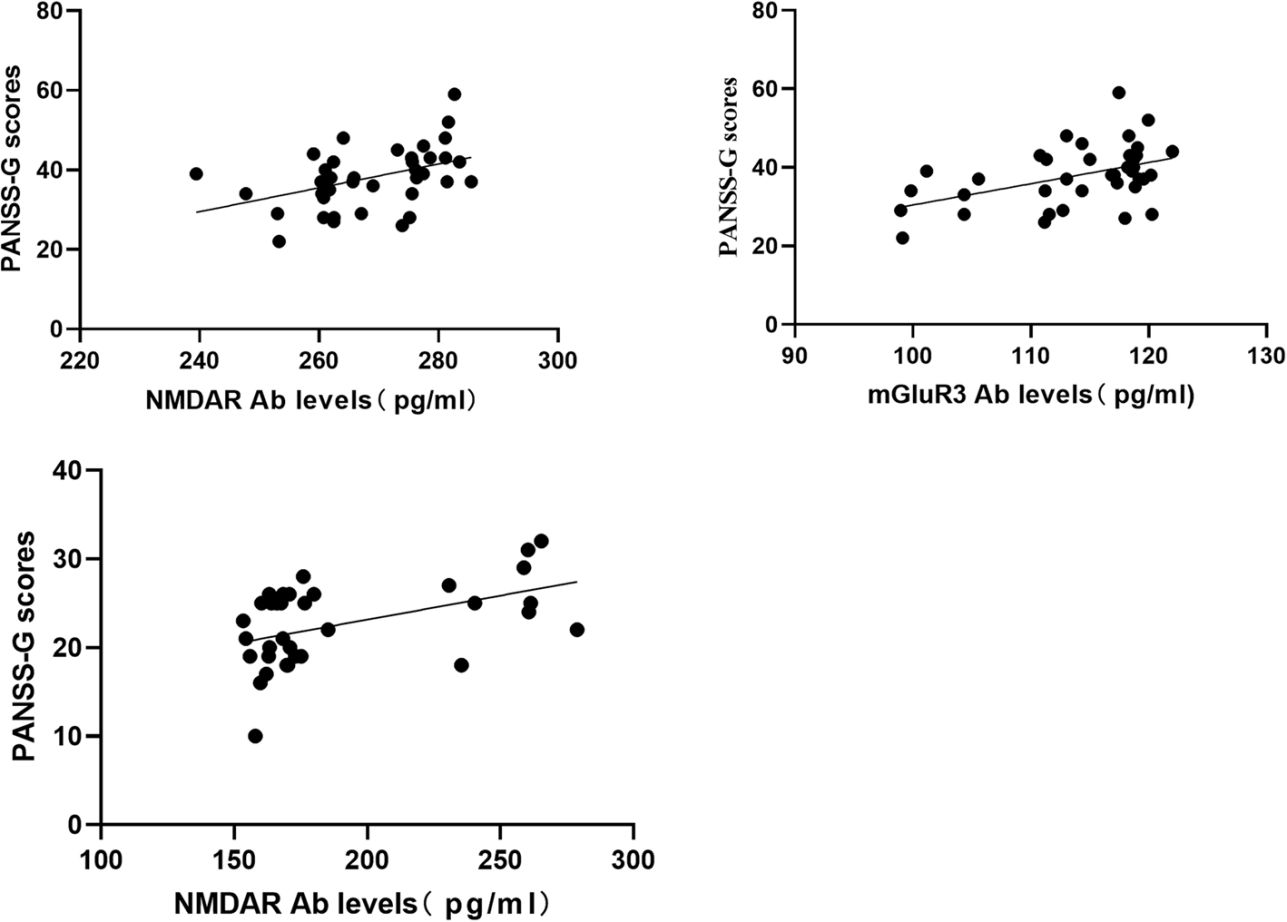
Correlations between serum anti-glutamate receptor antibody levels and PANSS-G scores in clozapine-naïve patients with treatment resistant schizophrenia. Abbreviations: NMDAR, N-methyl-D-aspartate Receptor; mGluR, Metabotropic glutamate receptor; Ab, antibody; PANSS-G, General Psychopathology scale

In the TRS-C group, no significant correlations were found between the anti-glutamate receptor antibody levels and PANSS scores (all ps > 0.05); in the TRS-NC group, PANSS-general sub-scores were positively correlated with anti-NMDAR antibody levels (*r* = 0.414, *p* = 0.009, FDR-corrected *q* = 0.072 > 0.05) and anti-mGluR3 antibody levels (*r* = 0.443, *p* = 0.005, FDR-corrected *q* = 0.08 > 0.05); in the In the nTRS group, general symptom scale scores were positively correlated with anti-NMDAR antibody levels (*r* = 0.438, *p* = 0.008, FDR-corrected *q* = 0.128 > 0.05) and anti-mGluR5 antibody levels (*r* = 0.389, *p* = 0.021, FDR-corrected *q* = 0.168 > 0.05) (Fig. 2). However, the above correlations failed to pass the FDR correction.

### Relationship between anti-glutamate receptor antibody levels and cognitive function

In the TRS-C group, no significant correlations were found between the levels of the four anti-glutamate receptor antibodies and MCCB scores (all ps > 0.05); in the TRS-NC group, anti-NMDAR antibody levels showed significant negative correlations with working memory scores (*r* = − 0.342, *p* = 0.033, FDR-corrected *q* = 0.352 > 0.05) and total scores (*r* = − 0.374, *p* = 0.019, FDR-corrected *q* = 0.608 > 0.05), anti-AMPAR antibody levels showed negative correlations with visual learning scores (*r* = − 0.373, *p* = 0.019, FDR-corrected *q* = 0.304 > 0.05) and total scores (*r* = − 0.320, *p* = 0.049, FDR-corrected *q* = 0.314 > 0.05), and anti-mGluR5 antibody levels showed negative correlations with visual learning scores(*r* = − 0.327, *p* = 0.042, FDR-corrected *q* = 0.336 > 0.05); in the NTRS group, anti-NMDAR antibody levels showed negative correlations with reasoning and problem solving scores (*r* = − 0.360, *p* = 0.034, FDR-corrected *q* = 0.544 > 0.05), and anti-mGluR5 antibody levels was negatively correlated with reasoning and problem solving scores(*r* = − 0.372, *p* = 0.028, FDR-corrected *q* = 0.896 > 0.05). However, all of these findings did not pass the FDR correction.

### Relationship between anti-glutamate receptor antibody levels and clozapine dose

We also explored the relationship between anti-glutamate receptor antibody levels and clozapine doses in the TRS-C group. No relationship between anti-glutamate receptor antibody levels and clozapine dose were identified.

## Discussion

The aim of our study was to compare serum levels of anti-glutamate receptor antibodies between patients with TRS and nTRS patients, and to investigate the impact of clozapine on antibody levels. Currently, there is limited research on anti-glutamate receptor antibody levels in TRS. Only one study directly measured serum anti-NMDAR antibody concentrations in TRS patients using ELISA, and our study’s results were consistent with that study, indicating that increased anti-NMDAR antibody levels are associated with antipsychotic drug resistance [33]. Additionally, this study measured the concentrations of several other serum anti-glutamate receptor antibodies for the first time, and the results were similar to those of anti-NMDAR antibodies, demonstrating that TRS patients have higher serum anti-glutamate receptor antibody levels compared to nTRS patients. Furthermore, the study found that TRS-C group patients had lower levels of anti-glutamate receptors, milder positive symptoms, and partially improved cognitive function compared to patients in the TRS-nC group. These findings collectively suggest that TRS may be linked to elevated levels of anti-glutamate receptor antibodies, and clozapine may alleviate clinical symptoms and cognitive deficits that are resistant to other antipsychotics by normalizing the levels of anti-glutamate receptor antibodies.

Extensive research has demonstrated the involvement of NMDAR dysfunction in the pathogenesis of schizophrenia, which is widely associated with psychotic symptoms and cognitive impairment [38]. Besides, a case has been reported that a female patient who had schizophrenia after remission of anti-NMDA receptor encephalitis and NMDA receptor antibody was not detected in the episodes, which inferred that NMDA receptor antibody might induce a long-term dysfunction on NMDA receptor and reduce the threshold for the onset of schizophrenia [39]. In addition to NMDAR dysfunction, AMPAR dysfunction mentioned in this study has been associated with selective attention and memory impairment in schizophrenia [40]. Activation of mGluR3 in the prefrontal cortex plays a crucial role in working memory and cognition [41], and genetic variations in GRM3, the gene encoding mGlu3, have been linked to schizophrenia and cognitive deficits [42]. The knockout of mGluR5 in mice has been shown to result in defects in prepulse inhibition, serving as a deficit model for schizophrenia [43]. Agonists targeting mGluRs have emerged as new candidates for antipsychotic drug development. Preclinical studies have demonstrated the therapeutic efficacy of both mGluR3 and mGluR5 agonists in schizophrenia, including the improvement of negative symptoms and cognitive deficits that are often unresponsive to current antipsychotics [44, 45]. Consistent with our hypotheses, TRS patients exhibit higher levels of various anti-glutamate receptor antibodies that disrupt glutamate receptor function, resulting in more severe symptoms and cognitive deficits compared to nTRS patients. In contrast, current antipsychotic drugs primarily target dopamine receptors and have limited effects on negative symptoms and cognitive impairment, which are significant contributors to treatment resistance.

However, current studies on the prevalence of anti-NMDAR antibodies in individuals with schizophrenia seem to show controversial results. Most of the studies on anti-NMDAR antibody detection showed negative results. Besides, there have been some researches investigating the prevalence of AMPA-type antibodies, however, no significant difference between patients and healthy controls in the prevalence was found [25, 30, 46]. This might be attributed to that the two main current methods, immunohistochemistry and cell-based assays, were relatively insensitive to human antibodies [47]. Consistent with our study, the previous studies using ELISA have found that higher anti-NMDA antibody levels in patients with TRS and other subtypes of schizophrenia compared with healthy individuals [32–34]. Hence, the development of more sensitive methods is urgent. In addition, although autoimmune mechanisms and other glutamate receptor dysfunction are crucial for the pathogenesis of schizophrenia, to our knowledge, the current studies on glutamate receptors and schizophrenia have mostly focused on NMDA-type. In this study, we emphasized that the types of anti-glutamate antibodies associated with schizophrenia were not limited to the NMDA-type. Indeed, there have been cases reported that some novel autoantibodies, such as glutamic acid decarboxylase 65 (GAD65) antibodies, were found in patients with schizophrenia [48]. Broadening the types of autoantibodies related to schizophrenia may be essential for clinical diagnosis and treatment.

The results of our study provide further evidence that clozapine may modulate the function of anti-glutamate receptors by reducing antibody levels, which could be one of the therapeutic mechanisms that distinguish clozapine from other antipsychotics. This finding is supported by a previous study that reported significantly lower immunoglobulin levels in the clozapine-treated group compared to the clozapine-naive group [36]. Moreover, this cross-sectional study revealed a significant association between the duration of clozapine treatment and the extent of reduction in serum IgG levels [36]. In recent years, there have been speculations regarding the link between clozapine and systemic autoimmune diseases (SAD), further supporting the possibility of this being a therapeutic mechanism of clozapine [36, 37, 49]. However, there is a limited number of studies, primarily consisting of isolated case reports, that demonstrate reduced autoantibody titers and symptom improvement in TRS patients with serum anti-NMDAR antibodies after clozapine administration [50].

The mechanism by which clozapine reduces anti-glutamate receptor antibody levels is currently unknown. It has been reported that dopamine D1 receptors are the main receptor type expressed by tonsillar B cells [51], and researchers have speculated that the underlying mechanism is related to the antagonistic effect of antipsychotic drugs on dopaminergic signaling in the germinal center [37]. However, this speculation also suggests that other antipsychotics should exhibit a stronger antagonistic effect than clozapine. Despite this, both previous studies and our current study consistently demonstrate lower antibody levels in the clozapine-treated group. Therefore, it is necessary to further explore the exact mechanism by which clozapine affects antibody levels and to investigate the effects of other antipsychotics on antibodies. In our study, no correlation was observed between clozapine dose and any of the anti-glutamate receptor antibody levels. This may be due to most patients in the clozapine-treated group receiving combination treatment with other antipsychotics, thereby limiting the ability to control for the effects of those medications. Additionally, individual variations in factors such as metabolism, smoking, gender, and concomitant use of other drugs can lead to different blood levels of clozapine, resulting in variations in the degree of antibody alteration [52, 53].

After applying the FDR correction, there was no evidence of a correlation between antibody concentrations and symptom severity or cognitive function in any of the three patient groups. This finding may be attributed to the exploratory nature of the study with its small sample size, which may render the multiple comparison correction excessively stringent. A similar study examining serum NMDAR antibodies in patients with TRS, nTRS, and treatment initiation schizophrenia (1. first-episode schizophrenia; 2. total duration of illness ≤3 years; and 3. within 2 weeks of initiation of antipsychotic medication), observed a correlation between antibody levels and symptom severity only in the treatment initiation schizophrenia group [33]. Long-term antipsychotic use also affects neurotransmitter mechanisms beyond glutamate, rendering the correlation between antibody levels and symptoms non-significant [33]. Additionally, generalized deficits in cognitive function in schizophrenia involve numerous possible mechanisms and other neurotransmitters, such as abnormalities in GABA, which may be associated with alterations in gamma oscillatory activity in the prefrontal cortex and subsequent working memory disturbances [54]. In the TRS-C group, no correlation was found between cognitive function, symptom severity, and the levels of the four antibodies. This may be due to the complex mechanism of action of clozapine, which not only affects glutamate antibody levels but also enhances the function of glutamate receptors, including NMDAR, through various mechanisms such as blocking glycine uptake, increasing glycine site occupancy, and interacting with mGluR5 receptors [55–58]. Furthermore, clozapine can also act on other receptors, including muscarinic, histamine, and serotonin receptors.

This study has certain limitations. Firstly, as a cross-sectional study, it is difficult to establish causal relationships. Due to the specific characteristics of the study population, the sample size is relatively small, and it was challenging to recruit three groups of participants who were matched in terms of illness duration, education level, and medication. Therefore, in data analysis, we employed included covariates rigorously to mitigate their influence. Additionally, the use of clozapine in clinical practice is relatively conservative, with patients being more inclined to choose higher doses of other antipsychotic medications or to add low doses of clozapine. Consequently, it was difficult to recruit patients who received clozapine monotherapy. The initiation of clozapine treatment is often delayed, and the participants had a longer illness duration, leading to several confounding factors that are difficult to exclude. Therefore, future studies should consider including healthy controls and patients with first-episode schizophrenia and conduct longitudinal research to further validate the results of this study.

## Conclusions

In summary, our study found a correlation between increased levels of anti-glutamate receptor antibodies and TRS, and the types of antibodies were not limited to the NMDA-type. Furthermore, we discovered that clozapine may have the ability to reduce these antibody levels in TRS patients. These findings provide supporting evidence for both the hypothesis of glutamatergic dysfunction and the neuroimmunological hypothesis. Additionally, our results propose that the superior efficacy of clozapine may be attributed, at least in part, to its effect on anti-glutamate receptor antibody levels. In order to gain a deeper understanding of the mechanism by which clozapine reduces antibody levels, large-scale longitudinal studies and research using animal models are essential. Moreover, schizophrenia researches related to novel antibody types, development of more sensitive methods, and treatment for abnormal antibody levels are urgently needed.

## Data Availability

The datasets used and analyzed during the current study are available from the corresponding author on reasonable request.
